# Genetic Biomarkers for ALS Disease in Transgenic SOD1^G93A^ Mice

**DOI:** 10.1371/journal.pone.0032632

**Published:** 2012-03-07

**Authors:** Ana C. Calvo, Raquel Manzano, Gabriela Atencia-Cibreiro, Sara Oliván, María J. Muñoz, Pilar Zaragoza, Pilar Cordero-Vázquez, Jesús Esteban-Pérez, Alberto García-Redondo, Rosario Osta

**Affiliations:** 1 Laboratorio de Genética Bioquímica (LAGENBIO-I3A), Aragon's Institute of Health Sciences (IACS), Facultad de Veterinaria, Universidad de Zaragoza, Zaragoza, Spain; 2 Unidad de ELA, Instituto de Investigación Hospital 12 de Octubre de Madrid, SERMAS, and Centro de Investigación Biomédica en Red de Enfermedades Raras (CIBERER U-723), Madrid, Spain; National Institutes of Health, United States of America

## Abstract

The pathophysiological mechanisms of both familial and sporadic Amyotrophic Lateral Sclerosis (ALS) are unknown, although growing evidence suggests that skeletal muscle tissue is a primary target of ALS toxicity. Skeletal muscle biopsies were performed on transgenic SOD1^G93A^ mice, a mouse model of ALS, to determine genetic biomarkers of disease longevity. Mice were anesthetized with isoflurane, and three biopsy samples were obtained per animal at the three main stages of the disease. Transcriptional expression levels of seventeen genes, *Ankrd1*, *Calm1*, *Col19a1*, *Fbxo32*, *Gsr*, *Impa1*, *Mef2c, Mt2, Myf5*, *Myod1*, *Myog*, *Nnt*, *Nogo A*, *Pax7*, *Rrad*, *Sln* and *Snx10*, were tested in each muscle biopsy sample. Total RNA was extracted using TRIzol Reagent according to the manufacturer's protocol, and variations in gene expression were assayed by real-time PCR for all of the samples. The Pearson correlation coefficient was used to determine the linear correlation between transcriptional expression levels throughout disease progression and longevity. Consistent with the results obtained from total skeletal muscle of transgenic SOD1^G93A^ mice and 74-day-old denervated mice, five genes (*Mef2c*, *Gsr*, *Col19a1*, *Calm1* and *Snx10*) could be considered potential genetic biomarkers of longevity in transgenic SOD1^G93A^ mice. These results are important because they may lead to the exploration of previously unexamined tissues in the search for new disease biomarkers and even to the application of these findings in human studies.

## Introduction

According to Biomarkers Definitions Working Group, a biomarker, which must be objectively measured and evaluated, is “an indicator of normal biological processes, pathogenic processes, or pharmacologic responses to a therapeutic intervention” as well as “an indicator of functional and structural changes in organs and cells”. Therefore, biomarkers can also be considered potential therapeutic molecular targets [1].

Amyotrophic Lateral Sclerosis (ALS) is one of the most common neurodegenerative disorders, and the search for molecular markers is increasing. This is especially true of the search for prognostic markers that might be involved in or promote the neurodegeneration process. These markers can then use to predict the outcome for a patient who is suffering from the disease. Because familial and sporadic ALS share clinical and pathological signs, understanding of the pathophysiological processes in familial ALS (FALS) would also provide a better understanding of the neurodegenerative mechanisms in sporadic ALS (SALS) [2]. FALS follows a predominantly autosomal dominant pattern; in SALS, genetic factors that occur sporadically contribute to its pathogenesis. In particular, mutations in the copper/zinc superoxide-dismutase-1 gene (*SOD1*) [3]–[5], Tar DNA-binding protein gene (*TARDBP*) and, most recently discovered, the DNA/RNA-binding proteins *FUS* (fused in sarcoma) or *TLS* (translocation in liposarcoma) produce the typical adult-onset ALS phenotype, suggesting that alterations in RNA processing may play a central role in ALS pathogenesis [6]–[13].

Interestingly, a wide range of molecules involved in different molecular pathways in ALS, such as excitotoxicity, inflammation and oxidative stress, have been described during the last three decades as possible biomarkers of the disease [14]–[23]. Many studies have been carried out to detect molecular markers for the diagnosis of ALS in tissues such as the brain, spinal cord, blood or cerebrospinal fluid (CSF).

Although skeletal muscle plays an important role in the neurophysiological diagnosis of ALS [24], [25], growing evidence supports the fact that it can be considered a primary target of ALS toxicity [22], [26], [27]. Under neurodegenerative conditions in ALS, muscle atrophy can result from lost connections to motor neurons in the neuromuscular junction; this loss of connections is likely due to an energetic deficit in the mutant muscle that leads to pathological conditions [27]. Consequently, skeletal muscle shares a direct connection with the nervous system and can clearly contribute to an alteration in the functional communication between muscle and nerve. Furthermore, the study of ALS markers in this tissue suggests that it may be possible to take muscle biopsy samples from live patients or animal models; however, this is not the case for brain and spinal cord samples. The availability of muscle biopsy samples might make it possible to carry out more accurate studies during disease progression, especially when markers of the disease have yet to be found.

The transgenic mice that have a G93A mutation in the SOD1 gene (SOD1^G93A^) are one of the best characterized animal models for ALS disease and present both clinical and pathological characteristics of ALS patients [14]. The aim of this study was to use this mouse model to search for potential genetic biomarkers of the disease that could be used to predict the animals' longevity based on their transcriptional expression during disease progression. Among the seventeen genes tested, only five, *Mef2c*, *Gsr*, *Col19a1*, *Calm1* and *Snx10*, may be considered potential genetic biomarkers of longevity in ALS disease as there was a significant linear correlation between their transcriptional profile during disease progression and the longevity of the animals.

## Results

### Significant variation in gene expression profiles in transgenic SOD1^G93A^ mice throughout disease progression

**Figure 1:**
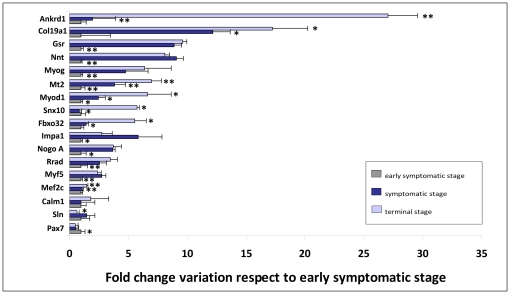
Transcriptional expression levels of the sixteen genes varied significantly throughout disease progression in transgenic SOD1^G93A^ mice. Representative graphs showing the fold change in transcriptional levels of the seventeen genes tested in the skeletal muscle of transgenic SOD1^G93A^ mice throughout disease progression with respect to the early symptomatic stage. Age-matched wild type mice were used as controls in each stage of the disease: early symptomatic (60 days, grey bar), symptomatic (90 days, blue bar) and terminal (120 days, pale blue bar) stages. The highest transcriptional expression levels were found in *Ankrd1* and *Col19a1*, which at the terminal stage were almost 27 and 18 times higher than those observed at the early symptomatic stage, respectively. A significant upregulation of transcriptional levels was found in all of the genes, except for *Calm1,* despite its irregular profile pattern throughout disease progression.

The profile expression pattern of the seventeen genes tested varied significantly throughout disease progression in the skeletal muscle of transgenic SOD1^G93A^ mice when compared to control, age-matched and wild type mice; the exception was *Calm1*, though this gene had an irregular expression pattern throughout the three disease stages. The transcriptional expression levels of *Sln*, another gene involved in calcium homeostasis, exhibited a significant variation in expression, indicating dysfunctions in calcium influx and calcium-induced endocytosis [28] (Figure 1). Moreover, *Ankrd1* and *Col19a1* produced the most significant difference in transcriptional expression patterns, especially at the terminal stage of the disease. This indicates there was an increase in muscle differentiation throughout disease progression that was prompted by an upregulation of *Col19a1* [29] and followed by an upregulation of *Myog*, *Myod1*, *Myf5* and *Mef2c*. Interestingly, the only myogenic regulatory factor that displayed a downregulation was *Pax7*, which would suggest that the myogenic potential and therefore the capacity for tissue repair were significantly diminished as neurodegeneration progressed; this finding is in accordance with the results of previous studies [30]. Significantly, this neurodegenerative progression also induced an upregulation in the transcriptional expression levels of *Ankrd1* and, to a lesser extent, *Nogo A*, *Fbxo32* and *Snx10*, which have previously found to be altered under degenerative conditions [27], [31]–[35]. Furthermore, we observed a significant upregulation in genes related to metabolic processes, *Impa1*, *Nnt*, *Rrad*, *Gsr* and *Mt2*, which may be altered under neurodegenerative conditions due to the uncoupled metabolic pathways they are involved in [36]–[39].

### Transcriptional expression of thirteen genes studied in muscle biopsies correlated in a linear fashion with longevity

The correlation study between gene expression profile and longevity focused on previously studied genes in transgenic SOD1^G93A^ and age-matched wild type mice. We hypothesized that those genes whose transcriptional expression yielded statistical significance during the progression of the disease in transgenic SOD1^G93A^ mice would be more likely to vary linearly through the disease stages and that they would correlate with longevity because they were involved in the neurodegenerative process of ALS. Therefore, the expression patterns of these genes could be used to predict longevity in transgenic SOD1^G93A^ mice.

The Pearson's correlation coefficients from each correlation study are shown in Table 1. The longevity of the mice ranged between 120 and 160 days. Importantly, we found that the transcriptional expression levels during disease progression of thirteen genes, *Myf5, Mef2c, Gsr, Myod1, Col19a1, Calm1, Myog, Snx10*, *Pax7, Impa1, Mt2, Ankrd1* and *Sln,* correlated significantly and negatively with longevity (Table 1). This negative correlation with longevity implies that the animals that survived longer displayed lower transcriptional levels of these genes during disease progression at the early symptomatic stage. Interestingly, unlike the other genes studied, *Mt2* and *Nnt*, displayed a positive correlation, which implied a longer survival when the transcriptional levels of *Mt2* and *Nnt* increased during the progression of the disease at the early symptomatic stage. The highest Pearson's correlation coefficients were found when testing *Myf5, Mef2c, Gsr, Myod1* and *Col19a1*, while the lowest coefficients were found for *Calm1, Myog, Snx10*, *Pax7, Impa1, Mt2, Ankrd1* and *Sln*, though they were still statistically significant. However, the transcriptional expression levels of *Nnt*, *Fbxo32*, *Rrad* and *Nogo A* did not display a significant correlation with longevity. These results suggest that an increase in myogenic potential may compensate for the muscle damage induced by the neurodegenerative progression of the disease, and therefore, the animals that survived longer may exhibit a higher regenerative capacity than the animals that survived for less time. It is possible that the animals with a shorter survival time had exhausted their myogenic regenerative capacity by overexpressing the myogenic precursors *Myf5*, *Mef2c*, *Myod1* and *Col19a1* at the early stages. This hypothesis is supported by the results shown in Figure 1 that indicated a significantly diminished myogenic potential at the terminal stage of 120 days, which is the shortest survival time among animals included in the muscle biopsy study.

**Table 1:** Pearson's correlation coefficients and statistical significance in the seventeen genes studied in the skeletal muscle biopsies of transgenic SOD1^G93A^ mice.

Gene	Pearson's correlation coefficient, r	p value
**MYF5**	−0,599	0,000
**MEF2C**	−0,552	0,000
**GSR**	−0,547	0,000
**MYOD1**	−0,527	0,000
**COL19A1**	−0,440	0,000
**CALM1**	−0,372	0,000
**MYOG**	−0,339	0,001
**SNX10**	−0,330	0,002
**PAX7**	−0,292	0,007
**IMPA1**	−0,265	0,014
**MT2**	0,256	0,018
**ANKRD1**	−0,244	0,001
**SLN**	−0,242	0,024
**NNT**	0,208	0,056
**FBXO32**	−0,188	0,080
**RRAD**	−0,166	0,129
**NOGO A**	−0,161	0,137

**Figure 2:**
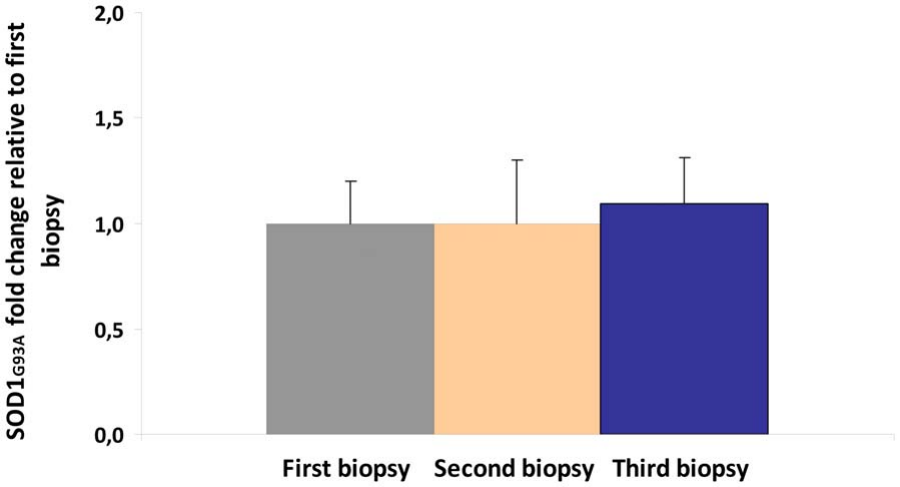
Transcriptional SOD1^G93A^ fold change in muscle biopsy samples. The transcriptional expression levels of SOD1^G93A^ were measured in all the samples obtained from the muscle biopsies corresponding to early symptomatic (first biopsy), symptomatic (second biopsy) and terminal stages (third biopsy). No statistical differences were found in SOD1^G93A^ levels along disease progression. SOD1^G93A^ fold change in the symptomatic and terminal stages was calculated respect to the relative expression found in all the muscle samples extracted at the early symptomatic stage.

Furthermore, expression levels of SOD1^G93A^ were measured as their significant variation could affect disease onset and progression. No statistical differences were found among the relative expression levels of SOD1^G93A^ in all the samples obtained from the three biopsies (Figure 2). This result confirmed the constant expression levels of SOD1^G93A^ in all the studied biopsies, suggesting that the neurodegenerative progress of the disease induced the different gene expression profiles in the studied genes.

From these results, we hypothesized that some of the genes that presented a significant correlation with longevity were involved in the denervation process that is present not only in ALS but also in all other known neurodegenerative disorders. To test this hypothesis, we used a denervation mouse model. To avoid false positive results, we tested all seventeen genes.

### The expression profiles of eleven genes varied significantly in the skeletal muscle of denervated mice

In this study, we identified the genes that were significantly altered in the skeletal muscle of denervated mice to differentiate the genes that are directly involved in the denervation process due to the disease from those that may be involved in the specific neurodegenerative process of ALS.

**Figure 3:**
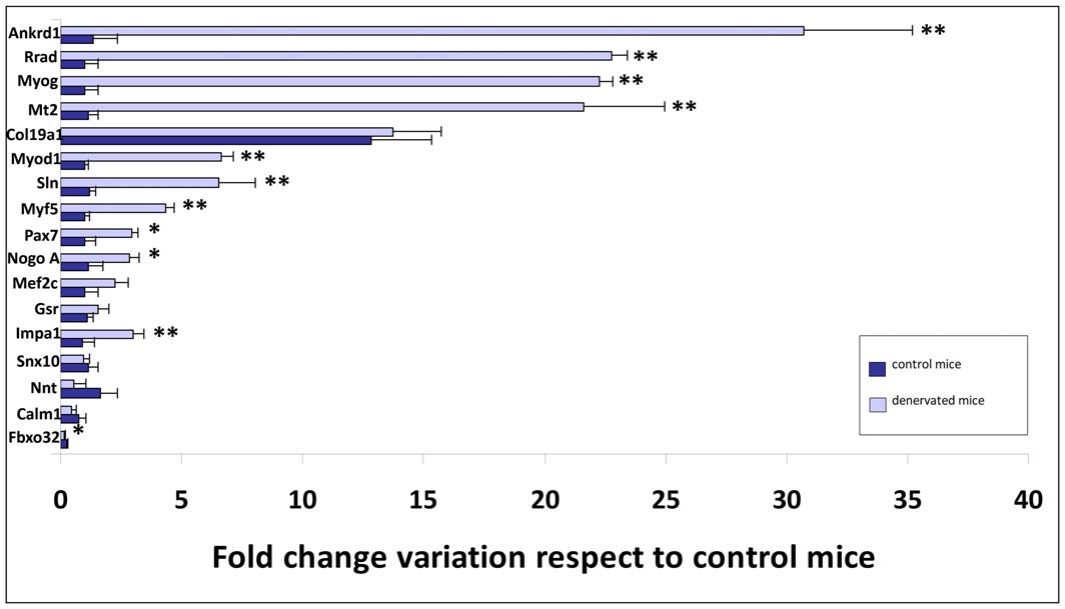
Eleven genes were related to the denervation process. Fold change variation in the transcriptional levels of seventeen genes in the skeletal muscle of 74-day-old denervated mice. Wild type mice aged for 60 days were used as controls. Among the seventeen genes tested, the transcriptional levels of *Ankrd1*, *Rrad*, *Myog, Mt2, Myod1*, *Sln*, *Myf5*, *Pax7*, *Nogo A, Impa1* and *Fbox32* varied significantly in denervated mice compared to control mice. *Fbox32* was the only gene that displayed a downregulated transcription level in denervated mice, which is probably due to its role in compensating for denervation-induced muscle atrophy via an internal mechanism.

Among the seventeen genes studied, eleven displayed significantly different transcriptional levels in denervated mice compared to age-matched wild type mice. Similar to the results obtained from transgenic SOD1^G93A^ mice during disease progression (Figure 1), the expression levels of *Ankrd1*, *Rrad*, *Myog, Mt2, Myod1*, *Sln*, *Myf5*, *Pax7*, *Nogo A* and *Impa1* were significantly increased in denervated mice; *Fbxo32*, however, displayed a decreased expression profile (Figure 3). Though the upregulation of *Fbxo32* has been associated with ALS [34], its downregulation appears to be related to an internal mechanism involved in reducing further loss of muscle proteins in denervation-induced muscle atrophy [40], which may explain our results. We also observed a downregulation of the transcriptional levels of *Pax7* in transgenic SOD1^G93A^ mice during disease progression (Figure 1), though in denervated mice, an overexpression of this gene was detected. This would suggest that mutant SOD1 toxicity might limit the capacity of mutant muscle to regenerate because the Pax7-expressing muscle progenitor pool in denervated muscles may remain less limited than in mutant muscles [30].

### Five potential genetic biomarkers of longevity in ALS

**Figure 4:**
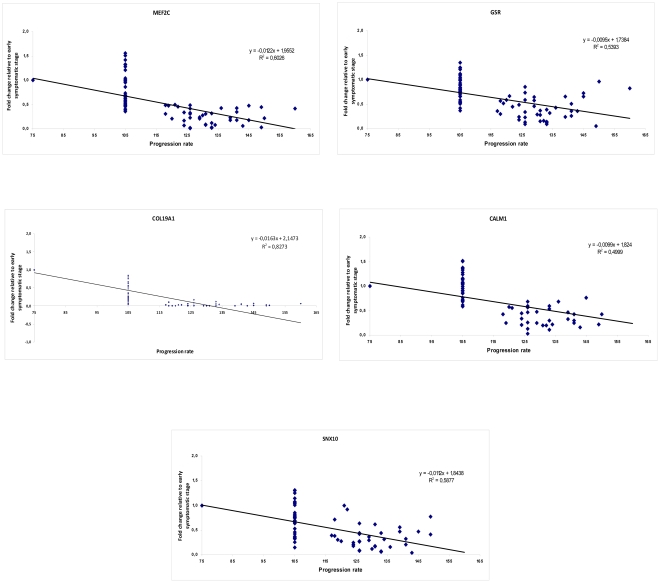
*Mef2c*, *Gsr*, *Col19a1*, *Calm1* and *Snx10 are potential genetic biomarkers of longevity in ALS*. Linear correlation graphs of five potential genetic biomarkers of longevity. The graph shows the linear relation between the fold change in transcriptional levels of these genes throughout disease progression in skeletal muscle biopsies at the early symptomatic stage and the longevity of the animals at the terminal stage. The transcriptional levels of these genes can predict longevity in transgenic SOD1^G93A^ mice.

The results observed under denervation conditions show that among the thirteen genes that displayed a significant correlation between transcriptional level expression and longevity during disease progression, five of them, *Mef2c*, *Gsr*, *Col19a1*, *Calm1* and *Snx10* may be considered potential biomarkers of longevity in ALS disease. The linear regression plots for these genes are shown in Figure 4. These results support the hypothesis that myogenic potential is significantly favored in animals that display longer survival and that an increase in transcriptional expression of these five genes makes it is possible to predict shorter longevity in transgenic SOD1^G93A^ mice.

## Discussion

Since Amyotrophic Lateral Sclerosis (ALS) was discovered and described in 1869 as a neurodegenerative disease characterized by motor neuron death and muscular atrophy, a wide range of biomarkers have been examined in the search for a therapeutic target. Although ALS shares altered molecular pathways with other neurodegenerative diseases, such as Alzheimer's, Huntington's or Parkinson's disease, there is an obvious need for specific ALS molecular markers that will allow an easier and earlier prognosis and/or diagnosis.

Because the skeletal muscle may be considered a primary target of ALS toxicity [22], [26], [27], we initially tested the gene expression profiles of seventeen genes using skeletal muscle from transgenic SOD1^G93A^ mice at different stages of disease progression. Interestingly, all the genes displayed significant changes in transcriptional expression throughout the three main stages of the disease, except for *Calm1*, though this was probably due to the high variability in its expression observed throughout the disease (Figure 1). An increase in the level of *Calm1* expression under degenerative conditions in mutant muscle, especially during the terminal stage, could activate expression of *Mef2c* and thereby activate a regenerative myogenic pathway. Similarly, increasing levels of *Col19a1*, *Mef2c*, *Myf5*, *Myog* and *Myod1* were also observed as a regenerative response to the muscle damage [41], [42]. However, the decreasing levels of *Pax7*, especially at the terminal stage (Figure 1), would indicate that myogenic regeneration was diminishing at the same time that muscle damage was increasing in this animal model [30]. According to our results, the upregulation of the transcriptional expression levels of *Ankrd1*, *Nogo A*, *Fbxo32* and *Snx10*, suggests that muscle damage increases throughout disease progression in transgenic SOD1^G93A^ mice [27], [31]–[35].

Furthermore, our results support those of a recent study in another mouse model for the disease, transgenic SOD1^G86R^ mice, which suggested that the aberrant expression of *Rrad* in response to oxidative stress may be pathologically relevant [38]. The increasing transcriptional levels of *Gsr* and *Mt2* in transgenic SOD1^G93A^ mice could activate the upregulation of *Rrad* expression, together with *Impa1* and *Nnt* (Figure 1), prompting an alteration of the muscle excitation-contraction coupling regulated by *Rrad* [38]. This alteration of the muscle could also be negatively affected by decreasing *Sln* expression levels, as *Sln* regulates relaxation-contraction cycles [43].

We hypothesized from these results that one or more of these genes, especially *Ankrd1* and *Col19a1*, which showed the highest significant upregulation during the terminal stage (Figure 1), might represent a potential prognostic biomarker of the disease. To elucidate this, biopsy muscle samples from a balanced number of male and female transgenic SOD1^G93A^ mice were obtained at three disease stages: the early symptomatic (75 days), symptomatic (105 days) and endpoint stages. In this last stage, the longevity of each animal was different, allowing us carry out a correlation study between longevity and the transcriptional expression profiles of each gene during disease progression.

Interestingly, the expression of twelve genes, *Myf5, Mef2c, Gsr, Myod1, Col19a1, Calm1, Myog, Snx10*, *Pax7, Impa1, Ankrd1* and *Sln,* during the early symptomatic stage of the disease correlated significantly and negatively with longevity; *Mt2* was the exception (Table 1). This negative correlation implies that the higher the expression level of these genes during the progression of the disease, the shorter the survival of the animal. Importantly, the *Myf5, Mef2c, Gsr, Myod1* and *Col19a1* genes displayed the highest Pearson's coefficients with longevity, suggesting that animals displaying a downregulation in expression of these genes at the early symptomatic stage may exhibit a higher regenerative capacity to compensate for muscle damage, as they survived longer than animals that overexpressed these genes. Therefore, because myogenic potential was significantly diminished at 120 days in transgenic SOD1^G93A^ mice (Figure 1), the ability to maintain this potential and compensate for muscle damage must closely relate to survival of the animal.

Additionally, *Mt2* and *Nnt* expression levels were positively correlated with longevity, significantly for *Mt2* expression, suggesting that the upregulation of the t expression levels of these genes during disease progression could be induced by the neurodegenerative progress of the disease. However, *Mt2* and *Rrad* have also been shown, potentially, to be deregulated after denervation in mouse skeletal muscle [38], [44]. Furthermore, it has been observed that the expression of *Nogo A* in ALS skeletal muscle promotes denervation in transgenic SOD1^G86R^ mice [32]. Because the process of denervation is crucial to inducing muscle atrophy in ALS, we tested the transcriptional expression of seventeen genes in the skeletal muscle of denervated mice to determine whether these genes are related to the denervation process itself; denervation is present in all known neurodegenerative disorders, including ALS.

Among the seventeen genes studied, the expression profile of eleven varied significantly in the skeletal muscle of 74-day-old denervated mice from that in age-matched wild type control mice (Figure 3). Our results support previously published studies that found significant variation in the expression levels of *Rrad*, *Mt2* and *Nogo A*, together with *Ankrd1*, *Myog, Myod1*, *Sln*, *Myf5*, *Pax7*, *Impa1* and *Fbxo32,* in denervated mice. The highest expression profiles were observed for *Ankrd1*, *Rrad*, *Myog* and *Mt2*, which were approximately 20 to 30 times higher than those of controls. This was especially significant for *Ankrd1* expression. These results, along with those of previous studies [30], [32], [38], [44], suggest that after 74 days under denervation conditions, skeletal muscle exhibits damage. This is evidenced by deregulated levels of *Ankrd1*, *Fbxo32, Nogo A* and *Rrad* causing induction of muscle differentiation and a subsequent increase in expression levels of *Myog, Myod1*, *Myf5*, *Pax7* and *Mef2c* (although this is not significant for *Mef2c*) or overexpression of *Mt2*, *Sln* and *Impa1* in response to increasing oxidative stress levels and alterations in calcium and glucose homeostasis, previously described as characteristics of ALS [22]. In particular, the downregulation of *Fbxo32* may suggest that an internal mechanism is involved in reducing further loss of muscle proteins during chronic degeneration [40]. Although they were not significant, the decrease in the expression levels of *Calm1*, *Nnt* and *Snx10* may also represent such a mechanism due to their role in catabolic and anabolic processes that lead to reduced muscle mass [40]. Similarly, the increase in expression of *Col19a1* and *Gsr*, though not significant, suggests that there may be concomitant activation of a second regulatory mechanism that acts as an effective scavenger of reactive oxygen species [45] or that favors skeletal myogenesis [29].

Taken together, these results along with the significant Pearson correlation coefficients observed in muscle biopsies suggest that *Mef2c*, *Gsr*, *Col19a1*, *Calm1* and *Snx10* may be considered potential genetic biomarkers of longevity in a mouse model of ALS (Figure 4), and the level of their expression in skeletal muscle may predict the longevity of transgenic SOD1^G93A^ mice.

In summary, the first step of the complex neurodegenerative process in ALS remains to be elucidated. However, studying the skeletal muscle as an ALS target tissue that is more accessible than other tissues, such as the spinal cord or CSF, opens the door to the discovery of new biomarkers that may lead to more accurate knowledge of the disease. Our studies suggest that the transcriptional levels of *Mef2c*, *Gsr*, *Col19a1*, *Calm1* and *Snx10* are closely related to the neurodegenerative process of ALS in the skeletal muscle, in such a way that they can predict longevity in a mouse model for the disease. These observations could help promote further studies exploring new tissues, and the translation of these results to human samples may lead to the discovery of new biomarkers and therefore new potential therapeutic targets.

## Materials and Methods

### Transgenic SOD1^G93A^ mice

Inbred B6SJL SOD1^G93A^ mice (The Jackson Laboratory, Bar Harbor, ME, USA) were used in this study because they provide a suitable ALS disease model. These mice carry a G93A mutation (substitution of Glycine to Alanine at residue 93) in the human gene superoxide dismutase 1 (SOD1). Hemizygous mutants, obtained by crossing a mutant male with a wild-type (WT) female, were used for all of the experiments. The offspring were identified by PCR amplification of DNA extracted from tail tissue as described in The Jackson Laboratory protocol for genotyping hSOD1 transgenic mice (http://jaxmice.jax.org/pub-cgi/protocols.sh?objtype=protocol,protocolid=523). The animals were housed in the Unidad Mixta de Investigación of the University of Zaragoza, in accordance with international guidelines for the use of laboratory animals. Food and water were available *ad libitum*. Routine microbiological monitoring did not reveal evidence of infection with common murine pathogens. All of the experimental procedures were approved by the ethics committees of our institutions and followed the international guidelines for the use of laboratory animals, particularly the guidelines for the preclinical in vivo evaluation of pharmacologically active drugs for ALS/MND.

### Search for gene targets as potential biomarkers of disease

Based on a previous microarray study of wild type and transgenic SOD1^G93A^ mice (data not shown) and previously published results suggesting several possible molecular markers in the skeletal muscle of an ALS mouse model [46], seventeen genes were tested as potential biomarkers of ALS disease (Table 2). Because skeletal muscle was the target tissue, the majority of these genes are involved in muscle physiology and differentiation. Some are involved in metabolic and anabolic processes. Neuromuscular junction dismantlement has been described as the primary pathogenic event in transgenic SOD1 mice [22]. Among the genes involved in this neurodegenerative process, cardiac ankyrin repeat domain 1 (*Ankrd1*) plays an important role in skeletal muscle plasticity and appears to be a general marker of muscle damage when it is upregulated [47], while reticulon 4 (*Rtn4*, also known as *Nogo A*) accelerates the progressive failure of motor neuron innervation [31], [48]–[50]. Other targets lead more directly to muscle atrophy when they are upregulated in muscle; these include F-box only protein (*Fbxo32*), which enhances proteolysis, and sorting nexin 10 (*Snx10*), which promotes vacuolization in mammalian cells [35], [40], [51].

**Table 2:** Functional role and related molecular pathway of the seventeen genes studied.

GENE NAME	SYMBOL	GENE ID	FUNCTION	MOLECULAR PATHWAY
ankyrin repeat domain 1 (cardiac muscle)	*Ankrd1*	107765	marker of muscle damage	
			muscle plasticity	
F-box only protein 32	*Fbxo32*	67731	promotes skeletal muscle atrophy	MUSCLE DAMAGE
			reduction of its gene expression levels in spinal cord injury disorders	
sorting nexin 10	*Snx10*	71982	regulation of endosome homeostasis	
paired box gene 7	*Pax7*	18509	muscle development	
myogenic differentiation 1	*Myod1*	17927	myogenesis, muscle differentiation	MUSCLE DIFFERENTIATION
myogenic factor 5	*Myf5*	17877	regulator of regenerative myogenesis and homeostasis, muscle regeneration	AND REGENERATION
myocyte enhancer factor 2C	*Mef2c*	17260	maintenance sarcomere integrity, muscle differentiation	
myogenin	*Myog*	17928	differentiation of muscle cells	
collagen, type XIX, alpha 1	*Col19a1*	12823	esophageal muscle development and function	MAINTENANCE MUSCLE INTEGRITY
reticulon 4	*NOGO A*	68585	inhibitor axonal regeneration	AND MUSCLE REINNERVATION
calmodulin 1	*Calm1*	12313	calcium signal modulator	
			endocitosis mediator at nerve terminal	CALCIUM HOMEOSTASIS
sarcolipin	*Sln*	66402	regulator calcium transport and muscle relaxation-contraction cycles	
inositol (myo)-1(or 4)-monophosphatase 1	*Impa1*	55980	inositol homeostasis	
			activated target of calbindin	
nicotinamide nucleotide transhydrogenase	*Nnt*	18115	glucose homeostasis	GLUCOSE METABOLISM
ras-related associated with diabetes	*Rrad*	56437	glucose tolerance and insuline sensitivity	
			regulation intracellular calcium signalling	
glutathione reductase	*Gsr*	14782	oxidative stress metabolism	
metallothionein 2	*Mt2*	17750	metal binding and free radical scavenging properties	OXIDATIVE STRESS
			oxidative stress metabolIsm, zinc homeostasis	

The dismantling of the neuromuscular junction is a sign of muscle denervation, which might be a consequence of skeletal muscle hypermetabolism. Hypermetabolism leads to a constant energy deficit in transgenic mice, which precedes amyotrophy and muscle denervation [52]. Furthermore, glucose metabolism and calcium homeostasis are altered in this animal model [27]. In particular, possible genes related to glucose metabolism include inositol (myo)-1(or 4)-monophosphatase 1 (*Impa1*), which plays an important role in motor coordination and is directly involved in glucose metabolism [36]; nicotinamide nucleotide transhydrogenase (*Nnt*), which leads to appropriate glucose homeostasis in these mice when it is downregulated [37]; and ras-related associated with diabetes (*Rrad*), which promotes altered lipid metabolism and deregulates glucose uptake [53]. Rrad is also involved in disease progression, insofar as the accumulation of reactive oxygen species is coincident with its upregulation [38]. In skeletal muscle, calmodulin 1 (*Calm1*) and sarcolipin (*Sln*) are needed to reach an adequate calcium influx, which is necessary to either maintain synaptic transmission in the neuromuscular junction, in the case of calmodulin1 [54], or to regulate muscle relaxation–contraction cycles, in the case of sarcolipin [43].

Oxidative stress has also been described in ALS [22]. In particular, glutathione reductase (*Gs*r) [39] and metallothionein 2 (*Mt2*) [55] represent the most studied enzymes involved in this process. In fact, their deficiency results in muscle atrophy and oxidative injury.

In contrast to degenerative processes, regenerative processes tend to compensate for the induced unbalance. In skeletal muscle, many genes are involved in regenerative pathways, either by maintaining the integrity of the tissue and favoring skeletal myogenesis, as is the case for collagen, type XIX, alpha 1 (*Col19a1*) [29], or by promoting muscle differentiation and regeneration, as is the case for the paired box gene (*Pax7*), myogenic differentiation 1 (*Myod1*), myogenic factor 5 (*Myf5*), myocyte enhancer factor 2C (*Mef2c*) and myogenin (*Myog*) [30], [56]–[60].

### Extraction of muscle samples in wild type and transgenic SOD1^G93A^ animals

Hemizygous SOD1^G93A^ mice and age-matched nontransgenic wild-type control mice at the early symptomatic, symptomatic and terminal stages (n = 10 transgenic SOD1^G93A^ mice and 10 wild type mice, balanced males and females, per stage) were used to study gene expression throughout disease progression. All of the animals were sacrificed by intraperitoneal (i.p.) injection of sodium pentobarbital (100 mg/Kg) following the guidelines from the report of the American Veterinary Medical Association Panel on Euthanasia, J. Am. Vet. Med. Assoc., 2007 (http://icwdm.org/Publications/pdf/ControlMethods/Euthanasia/AVMA2007report.pdf.

All surgical material was sterilized before dissection. After dissection, skeletal muscle of the hind limbs was immediately frozen in liquid nitrogen and stored at −80°C. Samples were collected by simple random sampling using the Statistical Package for the Social Sciences (SPSS) 15.0.

### Extraction of biopsies from skeletal muscle of transgenic SOD1^G93A^ mice

Forty-eight transgenic SOD1^G93A^ male and female mice were used to study the correlation of gene expression with longevity (n = 24 mice per sex). Three muscle biopsies from the *Gluteus superficialis* muscle were obtained per mouse, from a different hind limb each time, at three different ages that coincided with the early symptomatic stage (75 days), symptomatic stage (105 days) and terminal stage (endpoint age). This innovative procedure allowed the study of gene expression in the same animal; it was possible to keep the animal alive at different disease stages throughout the study because the *Gluteus superficialis* muscle is easily to reach and its manipulation does not prevent the mouse from moving properly after each surgery. All surgical material was sterilized before the extraction. Twenty minutes before starting the extraction, the analgesic Meloxicam 2 mg/kg (Metacam© AINES Cox-2) was administered subcutaneously to the animal. Once the gluteus superficialis muscle was localized, the zone was shaved and then disinfected with 70% alcohol and povidone iodide.

Each mouse was anesthetized by administration of isoflurane (4–5%) and a constant flux of 1.5–2% isoflurane maintained using a facemask. The lack of motor reflexes was confirmed before starting the surgery. The body temperature was maintained at a constant level with a thermal blanket, and a moisturizer gel (Lubrithal©) was applied on the eyes to prevent corneal damage during the extraction.

Once the animal was prepared for surgery, an incision of <1 cm was made on the zone where the gluteus superficialis is localized. The connective tissue around this muscle was carefully removed. A small biopsy of the muscle, with a weight of approximately 3 mg (≈1 mm^2^), was transferred to an eppendorf tube containing RNA*later*® solution (AM7021, Ambion, Madrid, Spain) to preserve the extracted tissue. The zone was closed with staples (EZ 9 mm clip) and rehydrated with physiological serum 0.9%, and a scar gel (Aloe vet©) was applied to promote cicatrisation of the wound. The mouse was placed back into its cage,, and it's body temperature was maintained using an infrared lamp until it awoke. The cage contained hydrated food and paper to facilitate the recovery of the animal.

For 24 hours following surgery, the mouse was tested two times per day to check coordination and to ensure the viability of the next biopsy. One week following surgery, the staples were removed. This methodology was repeated in each animal for the three selected stages so that the three biopsy samples were obtained from each mouse were of from the same kind of muscle [61]. The final biopsy sample was extracted at the endpoint for each animal. The mice were sacrificed when they were unable to right themselves within 30 s after being placed on their side; this point was considered the survival endpoint according to the guidelines for preclinical testing and colony management [62], [63].

### Extraction of muscle samples in denervated animals

Six male wild-type mice of the B6CLJ strain were anesthetized (pentobarbital 30 mg/kg, i.p.) at the age of 60 days, and muscle denervation was performed following the methodology described previously [30]. After surgical denervation, the animals were sacrificed by cervical dislocation at 74 days of age, and the gastrocnemius muscles were dissected and frozen immediately in liquid nitrogen.

### RNA extraction, synthesis of cDNA and real time PCR assay

RNA*later*® solution was removed from the biopsy samples, and total RNA was extracted using the RNeasy Micro Kit protocol (74004, Qiagen-Izasa, Barcelona, Spain), which included treatment with Dnase I solution to eliminate genomic DNA.

Tissue samples from the skeletal muscle of transgenic SOD1^G93A^, wild type, denervated, heterozygous and hemizygous SMA mice were pulverized in liquid nitrogen in a cold mortar. In this group of samples, total RNA was extracted using TRIzol Reagent according to the manufacturers' protocol (Invitrogen S.A., Prat de Llobregat, Spain). RNA was treated to eliminate genomic DNA using the Turbo DNA-*free*™ kit (AM1907, Ambion, Madrid, Spain).

RNA extracted from both groups of samples was processed for reverse transcription (RT) using the SuperScript™ First-Strand Synthesis System kit (12371-019, Invitrogen S.A., Prat de Llobregat, Spain). Gene expression variations in all of the samples were assayed by real-time PCR. PCR reactions were carried out in a StepOne™ Real-Time PCR System (4387925, Applied Biosystems, Madrid, Spain). Primer and probe mixtures for each gene of interest were supplied by Applied Biosystems (Madrid, Spain) (Table 3).

**Table 3:** Taqman® probe and primer mixtures used in gene expression assays.

NAME	GEN SYMBOL	ACCESION NUMBER	PROBE LOCATION	PART NUMBER	ORGANISM
Ankyrin repeat domain 1 (cardiac muscle)	Ankrd1	NM_013468.3	Exon 8–9	Mm00496512_m1	*Mus Musculus*
Calmodulin 1	Calm	NM_009790.4	Exon 2–3	Mm00486655_m1	*Mus Musculus*
Collagen, type XIX, alpha 1	Col19a1	NM_007733.2	Exon 2–3	Mm00483576_m1	*Mus Musculus*
F-box only protein 32	Fbxo32	NM_026346.2	Exon 5–6	Mm01207878_m1	*Mus Musculus*
Glutathione reductase	Gsr	NM_010344.4	Exon 12–13	Mm00833903_m1	*Mus Musculus*
Inositol (myo)-1(or 4)-monophosphatase 1	Impa1	NM_018864.5	Exon 7–8	Mm00497770_m1	*Mus Musculus*
Metallothionein 2	Mt2	NM_008630.2	Exon 3-3	Mm00809556_s1	*Mus Musculus*
Myocyte enhancer factor 2C	Mef2c	NM_025282.2	Exon 1–2	Mm00600423_m1	*Mus Musculus*
Myogenic differentiation 1	Myod1	NM_010866.2	Exon 1–2	Mm00440387_m1	*Mus Musculus*
Myogenic factor 5	Myf5	NM_008656.5	Exon 2–3	Mm00435125_m1	*Mus Musculus*
Myogenin	Myog	NM_008656.5	Exon 1–2	Mm00446194_m1	*Mus Musculus*
Nicotinamide nucleotide transhydrogenase	Nnt	NM_031189.2	Exon 5–6	Mm00435154_m1	*Mus Musculus*
Paired box gene 7	Pax7	NM_011039.2	Exon 4–5	Mm00834079_m1	*Mus Musculus*
Ras-related associated with diabetes	Rrad	NM_019662.2	Exon 2–3	Mm00451053_m1	*Mus Musculus*
Reticulon 4	Rtn4	NM_194052.2	Exon 2–3	Mm00445861_m1	*Mus Musculus*
Sarcolipin	Sln	NM_025540.2	Exon 1–2	Mm00481536_m1	*Mus Musculus*
Sorting nexin 10	Snx10	NM_001127349.1	Exon 1–2	Mm00511049_m1	*Mus Musculus*
Actin, beta, cytoplasmic	Actb (β-actin)	NM_008084.2	Exon 3	4352932E	*Mus Musculus*
Glyceraldehyde-3-phosphate dehydrogenase	Gapdh	NM_007393.1	Exon 6	4352933E	*Mus Musculus*
18S ribosomal RNA (18S rRNA)	18S	X03205.1	_	Hs99999901_s1	*Homo sapiens*
Superoxide dismutase 1	Sod1	NM_000454.4	Exon 2–3	Hs00916176_m1	*Homo sapiens*

Reactions were performed in a final volume of 5 µL with 1× TaqMan® Fast Universal PCR Master Mix (4352042, No AmpErase® UNG, Applied Biosystems, Madrid, Spain), 1× of the primer and TaqMan® MGB probe mix for each studied gene and 2 µL of 10× diluted cDNA per reaction. Three endogenous genes (18S rRNA, GAPDH, and β-actin) were used for normalization following the methodology described previously [64]. All reactions were performed in triplicate and all reaction efficiencies of the primer/probe sets were close to 100%. Thermal cycling parameters were as follows: incubation at 95°C, 20 seconds, and 40 cycles of 95°C for 1 second and 60°C for 20 seconds.

### Gene expression analysis in total skeletal muscle samples of transgenic SOD1^G93A^ and denervated mice

Changes in the RNA expression levels of each gene were studied in the skeletal muscles samples of mice, balanced for males and females. The corresponding ΔC_T_ values of each extracted sample were normalized with geometric media of the selected housekeeping genes as described above. Age-matched wild type mice were used as controls when studying the disease progression in transgenic SOD1^G93A^ mice. Sixty-day-old wild-type mice were used as controls when studying the expression patterns of the selected genes in denervated mice.

The ΔΔC_T_ method was used to determine relative changes in transcriptional expression. Statistical analyses of the data were performed using the fold change 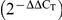 as previously described [64].

### Longevity correlation analysis of muscle biopsy samples

For each gene studied, the ΔΔC_T_ method was used to determine relative changes in gene expression, as previously described. Differences in the threshold cycles between the ΔC_T_ value at 75 days and at 105 days/the endpoint stage were calculated in all the tested genes for each animal under study. Because the first muscle biopsy sample was obtained when the animals showed early disease symptoms (75 days), ΔC_T 75 days_ was taken as a reference parameter to calculate the variation in the relative gene expression at the symptomatic (105 days) and endpoint stages with respect to the early symptomatic stage (75 days). Using this methodology, the corresponding fold changes were calculated using the equation 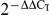. In this way, for each animal, three fold change values were obtained for each gene and were plotted to calculate the slope. The linear regression between each slope value for each animal and the age at which the animal died was studied. The Pearson correlation coefficient was used to determine the linear correlation between the slopes obtained for each studied gene and the longevity data.

### Statistical analysis

Statistical analyses were carried out using the non-parametric Mann-Whitney test to evaluate the variability in gene expression. The Pearson correlation coefficient was used to determine the linear correlation in wild type and transgenic SOD1^G93A^ mice with longevity throughout disease progression. All of the values were expressed as the mean ± S.E.M. The statistical significance threshold was set at p<0.05. The software used for the statistical analysis was SPSS 15.0.

## References

[1] Azuaje F (2010). Bioinformatics and Biomarker Discovery. “Omic” data analysis for personalized medicine.

[2] Siddique N, Siddique T (2008). Genetics of Amyotrophic Lateral Sclerosis.. Phys Med Rehabil Clin N Am.

[3] Hensley K, Mhatre M, Mou S, Pye QN, Stewart C (2006). On the relation of oxidative stress to neuroinflammation: lessons learned from the G93A-SOD1 mouse model of amyotrophic lateral sclerosis. Antioxid. Redox.. Signal.

[4] Cluskey S, Ramsden DB (2001). Mechanism of neurodegeneration in amyotrophic lateral sclerosis.. J Clin Pathol.

[5] Bruijn LI, Houseweart MK, Kato S, Anderson KL, Anderson SD (1998). Aggregation and motor neuron toxicity of an ALS-linked SOD1 mutant independent from wild-type SOD1.. Science.

[6] Wijesekera LC, Leigh PN (2009). Amyotrophic Lateral Sclerosis.. Orph J Rare Dis.

[7] Baek WS, Desai NP (2007). ALS: pitfalls in the diagnosis.. Pract Neurol.

[8] Turner MR, Kiernan MC, Nigel P (2009). Biomarkers in amyotrophic lateral sclerosis.. The Lancet.

[9] Kwiatkowski TJJ, Bosco DA, LeClerc AL, Tamrazian E, Vanderburg CR (2009). Mutations in the FUS/TLS Gene on Chromosome 16 Cause Familial Amyotrophic Lateral Sclerosis.. Science.

[10] Vance C, Rogelj B, Hortobágyi T, De Vos KJ, Nishimura AL (2009). Mutations in FUS, an RNA processing protein, cause Familial Amyotrophic Lateral Sclerosis type 6.. Science.

[11] Deng HX, Zhai H, Bigio EH, Yan J, Fecto F (2010). FUS-immunoreactive inclusions are a common feature in sporadic and non-SOD1 familial amyotrophic lateral sclerosis.. Ann Neurol.

[12] Liscic RM, Grinberg LT, Zidar J, Gitcho MA, Cairns NJ (2008). ALS and FTLD: two faces of TDP-43 proteinopathy.. European Journal of Neurology.

[13] Lagier-Tourenne C, Cleveland DW (2009). Rethinking ALS: The FUS about TDP-43.. Cell.

[14] Gurney ME, Pu H, Chiu AY, Dal Canto MC, Polchow CY (1994). Motor neuron degeneration in mice that express a human Cu,Zn superoxide dismutase mutation.. Science.

[15] Sumiyoshi H, Mor N, Lee SY, Doty S, Henderson S (2004). Esophageal muscle physiology and morphogenesis require assembly of a collagen XIX–rich basement membrane zone.. J Cell Biol.

[16] Mitsumoto H, Santella RM, Liu X, Bogdanov M, Zipprich J (2008). Oxidative stress biomarkers in sporadic ALS.. Amyotrophic Lateral Sclerosis.

[17] Ryberg H, Bowser R (2008). Protein biomarkers for amyotrophic lateral sclerosis.. Expert Rev Proteomics.

[18] Turner MR, Kiernan MC, Leigh PN, Talbot K (2009). Biomarkers in amyotrophic lateral sclerosis.. Lancet Neurol.

[19] Pradat PF, Dib M (2009). Biomarkers in amyotrophic lateral sclerosis: facts and future horizons.. Mol Diagn Ther.

[20] Pradat PF (2009). New biological and radiological markers in amyotrophic lateral sclerosis.. Presse Med.

[21] Wijesekera LC, Leigh PN (2009). Amyotrophic lateral sclerosis.. Orph J Rare Dis.

[22] Musaró A (2010). State of the art and the dark side of amyotrophic lateral sclerosis.. World J Biol Chem.

[23] de Carvalho M, Swash M (2011). Amyotrophic lateral sclerosis: an update.. Curr Opin Neurol.

[24] Douglass CP, Kandler RH, Shaw PJ (2010). An evaluation of neurophysiological criteria used in the diagnosis of motor neuron disease.. J Neurol Neurosurg Psychiatry.

[25] Burgunder JM, Schöls L, Baets J, Andersen P, Gasser T (2011). EFNS guidelines for the molecular diagnosis of neurogenetic disorders: motoneuron, peripheral nerve and muscle disorders.. Eur J Neurol.

[26] Dobrowolny G, Aucello M, Rizzuto E, Beccafico S, Mammucari C (2008). Skeletal muscle is a primary target of SOD1^G93A^-mediated toxicity.. Cell Metabolism.

[27] Dupuis L, Loeffler JP (2008). Sclérose latérale amyotrophique, junction neuromusculaire et _raline énergétique.. Medecine/Sciences.

[28] Babu GP, Bhupathy P, Timofeyev V, Petrashevskaya NN, Reiser PJ (2007). Ablation of sarcolipin enhances sarcoplasmic reticulum calcium transport and atrial contractility.. PNAS.

[29] Sumiyoshi H, Mor N, Lee SY, Doty S, Henderson S (2004). Esophageal muscle physiology and morphogenesis require assembly of a collagen XIX–rich basement membrane zone.. J Cell Biol.

[30] Manzano R, Toivonen JM, Oliván S, Calvo AC, Moreno-Igoa M (2011). Altered Expression of Myogenic Regulatory Factors in the Mouse Model of Amyotrophic Lateral Sclerosis.. Neurodegenerative Dis.

[31] Dupuis L, González de Aguilar JL, di Scala F, Rene F, de Tapia M (2002). Nogo provides a molecular marker for diagnosis of Amyotrophic Lateral Sclerosis.. Neurobiol Dis.

[32] Jokic N, González de Aguilar JL, Dimou L, Lin S, Fergani A (2006). EMBO Reports. http://dx.doi.org/10.1038/sj.embor.7400826.

[33] Nakamura K, Nakada C, Takeuchi K, Osaka M, Shomori K (2002–2003). Altered expression of cardiac ankyrin repeat protein and its homologue, ankyrin repeat protein with PEST and proline-rich region, in atrophic muscles in amyotrophic lateral sclerosis.. Pathobiology.

[34] Léger B, Vergani L, Sorarù G, Hespel P, Derawe W (2006). Human skeletal muscle atrophy in amyotrophic lateral sclerosis reveals a reduction in Akt and an increase in atrogin-1.. The FASEB J.

[35] Qin B, He M, Chen X, Pei D (2006). Sorting nexin 10 induces giant vacuoles in mammalian cells.. J Biol Chem.

[36] Berggård T, Szczepankiewicz O, Thulin E, Linse S (2002). Myo-inositol monophosphatase is an activated target of calbindin D28k.. J Biol Chem.

[37] Freeman HC, Hugill A, Dear NT, Ashcroft FM, Cox RD (2006). Deletion of nicotinamide nucleotide transhydrogenase: A new quantitive trait locus accounting for glucose intolerance in C57BL/6J mice.. Diabetes.

[38] Halter B, Gonzalez de Aguilar JL, Rene F, Petri S, Fricker B (2010). Oxidative stress in skeletal muscle stimulates early expression of Rad in a mouse model of amyotrophic lateral sclerosis.. Free Rad Biol Med.

[39] Dudley RWR, Khairallah M, Mohammed S, Lands L, Des Rosiers C (2006). Dynamic responses of the glutathione system to acute oxidative stress in dystrophic mouse (mdx) muscles.. Am J Physiol Regul Integr Comp Physiol.

[40] Léger B, Sense R, Al-Khodairy AW, Dériaz O, Gobelet C (2009). Atrogin-1, murf1, and foxo, as well as phosphorylated gsk-3β and 4e-bp1 are reduced in skeletal muscle of chronic spinal cord–injured patients.. Muscle & Nerve.

[41] Borselli C, Storrie H, Benesch-Lee F, Shvartsman D, Cezar C (2010). Functional muscle regeneration with combined delivery of angiogenesis and myogenesis factors.. PNAS.

[42] Sakuma K, Yamaguchi A (2010). The functional role of calcineurin in hypertrophy, regeneration, and disorders of skeletal muscle.. J Biomed Biotech.

[43] Vasu VT, Ott S, Hobson B, Rashidi V, Oommen S (2009). Sarcolipin and ubiquitin carboxy-terminal hydrolase 1 mRNAs are over-expressed in skeletal muscles of *α*-tocopherol deficient mice.. Free Radic Res.

[44] Magnusson C, Svensson A, Christerson U, Tågerud S (2005). Denervation-induced alterations in gene expression in mouse skeletal muscle.. Eur J Neurosci.

[45] Limon-Pacheco JH, Gonsebatt ME (2010). The glutathione system and its regulation by neurohormone melatonin in the central nervous system.. Cent Nerv Syst Agents Med Chem.

[46] Gonzalez de Aguilar JL, Niederhauser-Wiederkehr C, Halter B, de Tapia M, di Scala F (2008). Gene profiling of skeletal muscle in an amyotrophic lateral sclerosis mouse model.. Physiol Genomics.

[47] Laure L, Suel L, Roudaut C, Bourg N, Ouali A (2009). Cardiac ankyrin repeat protein is a marker of skeletal muscle pathological remodelling.. FEBS J.

[48] Fergani A, Dupuis L, Jokic N, Larmet Y, de Tapia M (2005). Reticulons as markers of neurological diseases: focus on amyotrophic lateral sclerosis.. Neurodegen Dis.

[49] Pradat PF, Bruneteau G, Gonzalez de Aguilar JL, Dupuis L, Jokic N (2007). Muscle Nogo-A expression is a prognostic marker in lower motor neuron syndromes.. Ann Neurol.

[50] Yan R, Shi Q, Hu X, Zhou X (2006). Reticulon proteins: emerging players in neurodegenerative diseases.. Cell Mol Life Sci.

[51] Lagirand-Cantaloube J, Cornille K, Csibi A, Batonnet-Pichon S, Leibovitch MP (2009). Inhibition of atrogin-1/MAFbx mediated MyoD proteolysis prevents skeletal muscle atrophy in vivo.. PLos One.

[52] Dupuis L, Loeffler JP (2009). Neuromuscular junction destruction during amyotrophic lateral sclerosis: insights from transgenic mice.. Curr Op Pharm.

[53] Ilany J, Bilan PJ, Kapur S, Caldwell JS, Patti ME (2006). Overexpression of Rad in muscle worsens diet-induced insulin resistance and glucose intolerance and lowers plasma triglyceride level.. PNAS.

[54] Wu XS, McNeil BD, Xu J, Fan J, Xue L (2009). Ca2+ and calmodulin initiate all forms of endocytosis during depolarization at a nerve terminal.. Nat Neurosci.

[55] DeRuisseau LR, Recca DM, Mogle JA, Zoccolillo M, DeRuisseau KC (2009). Metallothionein deficiency leads to soleus muscle contractile dysfunction following acute spinal cord injury in mice.. Am J Physiol Regul Integr Comp Physiol.

[56] White RB, Ziman MR (2008). Genome-wide discovery of Pax7 target genes during development.. Physiol Genomics.

[57] Zhao P, Caretti G, Mitchell S, McKeehan WL, Boskey AL (2006). Fgfr4 is required for effective muscle regeneration *in vivo*: Delineation of a MyoD-Tead2-Fgfr4 transcriptional pathway.. J Biol Chem.

[58] Gayraud-Morel B, Chrétien F, Flamant P, Gomès D, Zammit PS (2007). A role for the myogenic determination gene *Myf5* in adult regenerative myogenesis.. Develop Biol.

[59] Dodou E, Xu SM, Black BL (2003). *Mef2c* is activated directly by myogenic basic helix-loop-helix proteins during skeletal muscle development in vivo.. Mechanism Develop.

[60] Cao Y, Kumar RM, Penn BH, Berkes CA, Kooperberg C (2006). Global and gene-specific analyses show distinct roles for Myod and Myog at a common set of promoters.. The EMBO J.

[61] Murillo S, Calvo AC, Osta R (2009). Biopsia muscular en ratón..

[62] Ludolph A, Bendotti C, Blaugrund E, Chio A, Greensmith L (2010). Guidelines for preclinical animal research in ALS/MND: A consensus meeting.. Amyotrophic Lateral Sclerosis.

[63] Leitner M, Menzies S, Lutz C (2010). Working with ALS Mice. Guidelines for preclinical testing & colony management.

[64] Calvo AC, Moreno-Igoa M, Manzano R, Ordovás L, Yagüe G (2008). Determination of protein and RNA expression levels of common housekeeping genes in a mouse model of neurodegeneration.. Proteomics.

